# An Acute Presentation of Epstein-Barr Virus (EBV) Infection in an Immunocompromised Gentleman

**DOI:** 10.7759/cureus.33036

**Published:** 2022-12-28

**Authors:** Shaumick Bhattacharjee, Abuobeida Ali, Saber Sayed, Tahmina Siyal, Tapas Das

**Affiliations:** 1 Gastroenterology, Peterborough City Hospital, Peterborough, GBR

**Keywords:** gastroenterology hepatology, primary ebv infection, immunosuppression, ulcerative colitis (uc), ebv- associated hepatitis

## Abstract

Epstein-Barr virus (EBV) is a relevant cause of many clinical manifestations with a range of malignant and non-malignant presentations. This is particularly important to consider in immunosuppressed individuals. We present a case of a 36-year-old individual with ulcerative colitis who was in remission whilst taking mercaptopurine. The patient presented with weight loss, night sweats, and significant laboratory serum abnormalities on monitoring. Relevant investigations into his presentation ruled out a malignant feature, but his serology confirmed infection with EBV with the spread of infection to the liver and bone marrow. Overall, we identify a notable yet relevant clinical expression of EBV infection in the context of an immunosuppressed individual.

## Introduction

Epstein-Barr virus (EBV) is part of the human herpes virus family. It is a widespread infection in the human population, particularly in children under five years old, albeit mostly with no significant clinical manifestation [1]. In adults, it manifests mainly in the form of infectious mononucleosis (IM) or, more sinisterly, lymphoma-type malignancies [2]. Symptomatic acute IM typically involves patients complaining of fever, swollen neck lymph nodes, and a sore throat, which presents after an incubation of four to seven weeks [3]. Indeed, an increase in IM detection has occurred in the UK, suggesting the effective spread of EBV, with increasing EBV antibody detection in the population observed [4]. Normally, a self-limiting infection and the clinical format of its presentation in immunosuppressed individuals are less reported. A literature search identified accounts of EBV-induced hepatitis, but they normally present with a prodrome of infectious mononucleosis [5]. Although some reports highlight a phenomenon of hepatitis in the absence of other presentations, this was indeed a rarity [6-7]. We report a case that showcases a manifestation of acute EBV infection in an individual undergoing immunosuppression to control his background ulcerative colitis, with no other manifestations/prodromes identified.

## Case presentation

A 36-year-old gentleman with a background of left-sided ulcerative colitis, diagnosed in 2017 via colonoscopy and maintained in remission with mercaptopurine, had been suffering from self-reported continuing nausea, unintentional weight loss, and night sweats. At his regular follow-up, he discussed his symptoms with the inflammatory bowel disease (IBD) nurses. They had identified a worrying uptrend in his serum liver function tests (LFTs) and a downtrend in serum WBC and platelet counts. His history prior to the presentation included ulcerative colitis in remission. His remission was trialed with azathioprine in March 2018 after establishing negative viral serology concurrently with a maximum dose of mesalazine. Unfortunately, this was not tolerated, and after an acute flare in April 2018, he achieved remission with mercaptopurine. Additionally, he had recently been identified to be suffering from rheumatoid arthritis (RA) under the management of the rheumatology team.

Upon presentation to the acute medical unit, the examination was unremarkable. Admission investigations included cytomegalovirus (CMV), EBV, hepatitis E (HepE), and Parvovirus serology, along with hepatitis A, B, and C. A routine full blood count (FBC), urea and electrolytes (U&E’s), and LFTs were assessed again.

Investigations

**Figure 1:**
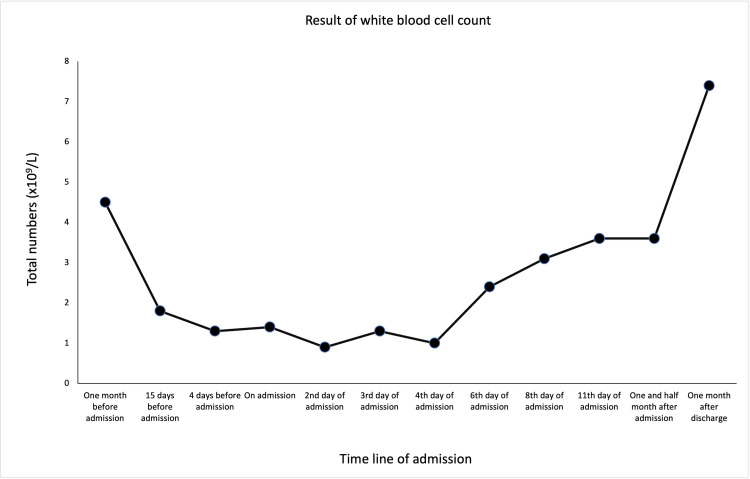
The trend of serum WBC count prior to admission following post-discharge.

**Figure 2:**
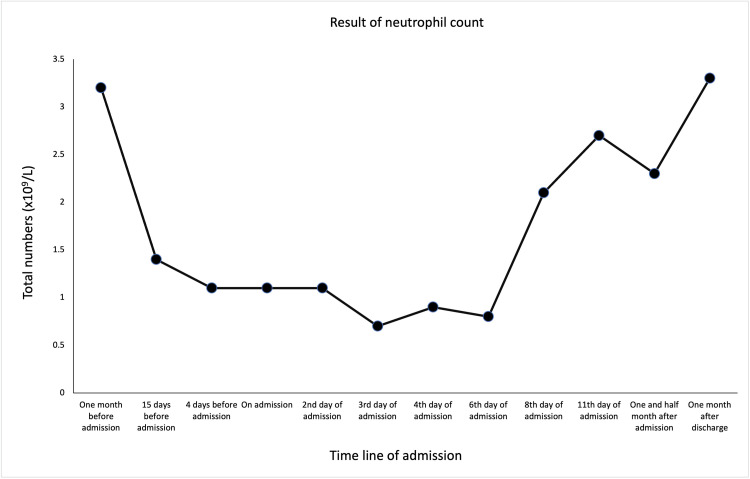
The trend of neutrophil count prior to admission following post-discharge.

**Figure 3:**
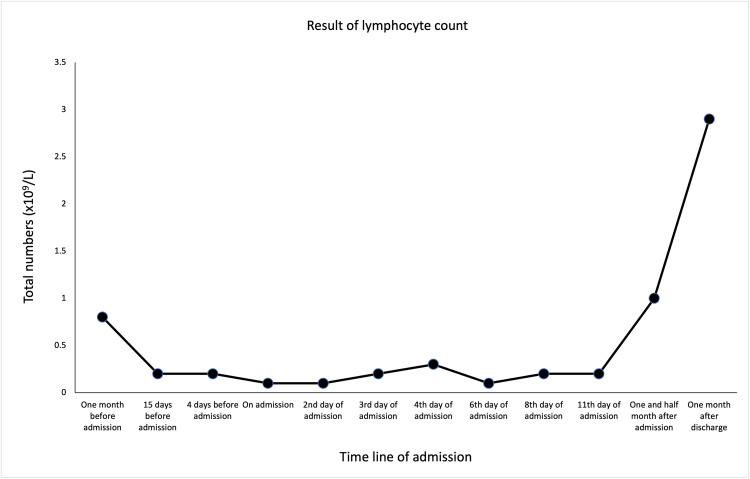
The trend of serum lymphocyte count prior to admission following post-discharge.

**Figure 4:**
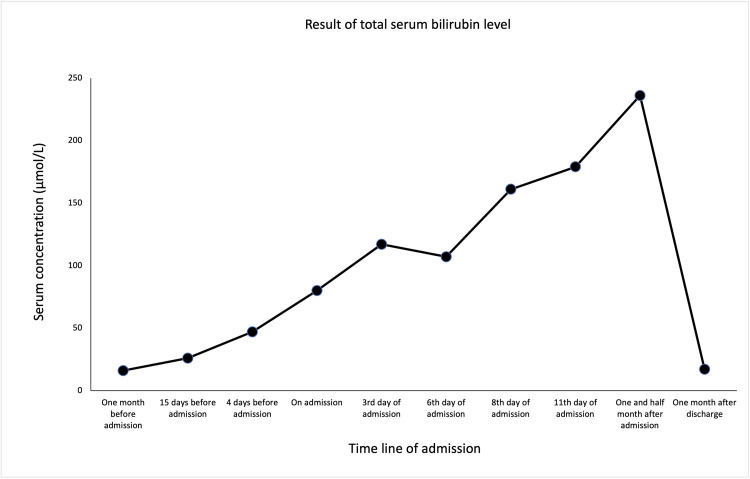
The trend of serum total bilirubin levels prior to admission following post-discharge.

The blood results, showcased in the following figures, showed rapidly worsening cytopenia (Figures 1-3), LFTs (Figure 4), and high ferritin. With the above presentation, the initial impression was of mercaptopurine-induced cytopenia with deranged LFTs. The initial serology screen showed that the patient was negative for hepatitis B, C (Hep B, C ) and associated liver autoantibodies, including anti-liver kidney microsomal (LKM) and anti-smooth muscle antibody (SMA). Interestingly, CMV IgM, EBC viral capsid (VC) IgM, and IgG were detected, while EBV nuclear antigen (EBNA) IgG was negative. However, the results were reported to have been a false positive due to the patient having a background of RA. In addition, infective screening for COVID-19 was negative.

While an inpatient, the gentleman had started displaying pyrexia and tachycardia and was commenced on empirical antibiotics although having an appropriate septic screen carried out. With the concern of the patient getting generally unwell, an ultrasound of the abdomen was carried out showed splenomegaly, free fluid, and heterogenous mass within the gallbladder suggestive of possible biliary sludge.

Diagnosis and management

The patient was commenced on management for neutropenic sepsis and anti-fungal cover with a view to immunosuppression while also commenced on granulocyte colony-stimulating factor (G-CSF). On the ninth day of his inpatient stay, it was observed that he had developed bilateral axillary, inguinal, and unilateral cervical lymphadenopathy. The patient continued suffering from pyrexial episodes, despite antibiotic escalation to meropenem. The patient subsequently underwent a biopsy of both liver and bone marrow. This finally ruled out lymphoma but suggested an appearance consistent with EBV infection. Definitive management involved the withdrawal of immunosuppression with mercaptopurine, with subsequent improvement in his overall WBC count (Figure 1) as well as neutrophils (Figure 2) and lymphocytes (Figure 3). His serum bilirubin levels (Figure 4) were observed post-discharge.

## Discussion

Opportunistic infections are commonplace in individuals undergoing immunosuppressive therapy, with EBV being a well-known cause of clinical presentation [1,8]. EBV is a gamma family human herpes virus and is one of the most widespread infections worldwide, with a largely asymptomatic course [3,9]. However, when the host immune response to virus balance is disrupted, there is a risk of significant known complications of EBV infection. These include infectious mononucleosis, hepatitis, multiple sclerosis, and lymphoproliferative cancers (Hodgkin’s lymphoma, Burkitt’s lymphoma, etc.). Clinically, patients may display EBV infections that are usually spread through saliva, with subsequent infiltration into either B cells or epithelial cells and a resultant lifelong infection while normally alternating between two stages of infection: latent or lytic stage [8-10]. EBV normally results in self-limiting infections, mainly presenting as infectious mononucleosis, characterized as fever, malaise, tonsillitis, and lymphadenopathy. The significant complication mainly known to result from EBV is in the form of malignancy. There are limited findings or reports that detail alternative clinical presentations of EBV infection [2]. We believe that, in our case, the lytic stage of EBV can be held responsible for the presentation, with symptoms resulting from the general expansion of the viral numbers along with systemic inflammatory symptoms.

Blood investigations that can guide the way of an acute EBV infection are in the format of LFTs, with derangement providing a hepatic picture [11]. Additionally, this is to be confirmed with appropriate serology, as we have highlighted in our results. This would involve measuring the serology of the viral capsid together with the nuclear antigen. An acute infection is signified by the presence of at least IgM antibodies to the viral capsid but has to be added with a negative nuclear antigen result. A previous infection is normally understood to be in the presence of IgG antibodies to the nuclear antigen. However, clinical symptom generation from EBV-induced hepatitis is not well known, with only a few reports noting this [6-7].

Our case highlights the value of considering EBV infection in a deranged hepatic picture in an immunocompromised individual. Despite the resolution of his infection after returning to an immunocompetent stage, there will have to be a balance reached, particularly by controlling his background ulcerative colitis. Limited studies have identified appropriate antiviral therapy in managing acute EBV infections. Furthermore, the impact of being at increased risk of cancer in the future deems an individual to be screened in the future [12-14]. Multiple studies have identified that a high viral count, seen in the lytic stage of infection, is a high-grade prognostic value in predicting cancer development [15,16].

## Conclusions

In conclusion, we report a case of an individual who had suffered from an acute EBV infection following recent immunosuppression to maintain remission of his background ulcerative colitis. It highlights the value of early viral screening in patients who are being treated in the same way, extending to patients being managed for rheumatological conditions. Additionally, such patients should be considered for a screening pathway due to the high risk of developing cancer in the future.
